# Supplementary material for: Validation of a deep learning, value-based care model to predict mortality and comorbidities from chest radiographs in COVID-19
Source: PLOS Digit Health. 2022 Aug 1;1(8):e0000057. doi: 10.1371/journal.pdig.0000057 (PMC9931278; doi:10.1371/journal.pdig.0000057)
Supplement: S1 File — Pyrros A, Rodríguez-Fernández JM, Borstelmann SM, Gichoya JW, Horowitz JM, Fornelli B, Siddiqui N, Velichko Y, Koyejo Sanmi O, Galanter W. Detecting Racial/Ethnic Health Disparities Using Deep Learning From Frontal Chest Radiography. J Am Coll Radiol. 2022 Jan;19(1 Pt B):184–191. doi: 10.1016/j.jacr.2021.09.010. Erratum in: J Am Coll Radiol. 2022 Mar;19(3):403. PMID: 35033309; PMCID: PMC8820271. (PDF) [file pdig.0000057.s001.pdf]

Check for updates

Detecting Racial/Ethnic Health Disparities Using Deep Learning From Frontal Chest Radiography

SA-CME

Ayis Pyrros, MD[a], Jorge Mario Rodríguez-Fernández, MD[b], Stephen M. Borstelmann, MD[c], Judy Wawira Gichoya, MD[d], Jeanne M. Horowitz, MD[e], Brian Fornelli, MS[f], Nasir Siddiqui, MD[a], Yury Velichko, PhD[e], Oluwasanmi Koyejo Sanmi, PhD[g], William Galanter, MD, PhD[h]

Credits awarded for this enduring activity are designated "SA-CME" by the American Board of Radiology (ABR) and qualify toward fulfilling requirements for Maintenance of Certification (MOC) Part II: Lifelong Learning and Self-assessment. To access the SA-CME activity visit https://cortex.acr.org/Presenters/CaseScript/CaseView?Info=6WPmHu%2f41qpm2wOzNcANIMo02PvBiyE6wrPDXWbqquk%253d. SA-CME credit for this article expires January 3, 2025.

Abstract

Purpose: The aim of this study was to assess racial/ethnic and socioeconomic disparities in the difference between atherosclerotic vascular disease prevalence measured by a multitask convolutional neural network (CNN) deep learning model using frontal chest radiographs (CXRs) and the prevalence reflected by administrative hierarchical condition category codes in two cohorts of patients with coronavirus disease 2019 (COVID-19).

Methods: A CNN model, previously published, was trained to predict atherosclerotic disease from ambulatory frontal CXRs. The model was then validated on two cohorts of patients with COVID-19: 814 ambulatory patients from a suburban location (presenting from March 14, 2020, to October 24, 2020, the internal ambulatory cohort) and 485 hospitalized patients from an inner-city location (hospitalized from March 14, 2020, to August 12, 2020, the external hospitalized cohort). The CNN model predictions were validated against electronic health record administrative codes in both cohorts and assessed using the area under the receiver operating characteristic curve (AUC). The CXRs from the ambulatory cohort were also reviewed by two board-certified radiologists and compared with the CNN-predicted values for the same cohort to produce a receiver operating characteristic curve and the AUC. The atherosclerosis diagnosis discrepancy, Δ_{vasc}, referring to the difference between the predicted value and presence or absence of the vascular disease HCC categorical code, was calculated. Linear regression was performed to determine the association of Δ_{vasc} with the covariates of age, sex, race/ethnicity, language preference, and social deprivation index. Logistic regression was used to look for an association between the presence of any hierarchical condition category codes with Δ_{vasc} and other covariates.

Results: The CNN prediction for vascular disease from frontal CXRs in the ambulatory cohort had an AUC of 0.85 (95% confidence interval, 0.82-0.89) and in the hospitalized cohort had an AUC of 0.69 (95% confidence interval, 0.64-0.75) against the electronic health record data. In the ambulatory cohort, the consensus radiologists' reading had an AUC of 0.89 (95% confidence interval, 0.86-0.92) relative to the CNN. Multivariate linear regression of Δ_{vasc} in the ambulatory cohort demonstrated significant negative associations with non-English-language preference ($\beta = -0.083$, $P < .05$) and Black or Hispanic race/ethnicity ($\beta = -0.048$, $P < .05$) and positive associations with age ($\beta = 0.005$, $P < .001$) and sex ($\beta = 0.044$, $P < .05$). For the hospitalized cohort, age was also significant ($\beta = 0.003$, $P < .01$), as was social deprivation index ($\beta = 0.002$, $P < .05$). The Δ_{vasc} variable (odds ratio [OR], 0.34), Black or Hispanic race/ethnicity (OR, 1.58), non-English-language preference (OR, 1.74), and site (OR, 0.22) were independent predictors of having one or more hierarchical condition category codes ($P < .01$ for all) in the combined patient cohort.

[a]Department of Radiology, Duly Health and Care, Hinsdale, Illinois.
[b]Department of Neurology, University of Illinois at Chicago, Chicago, Illinois.
[c]Department of Radiology, University of Central Florida, Orlando, Florida.
[d]Department of Radiology, Emory University, Atlanta, Georgia.
[e]Department of Radiology, Northwestern Memorial Hospital, Northwestern University, Chicago, Illinois.
[f]Anthem Inc, Chicago, Illinois.
[g]Department of Computer Science, University of Illinois at Urbana-Champaign, Urbana-Champaign, Illinois.
[h]Department of Medicine, University of Illinois at Chicago, Chicago, Illinois.

Corresponding author and reprints: Ayis Pyrros, MD, Department of Radiology, Duly Health and Care, 40 S Clay Street, Hinsdale, IL 60521; e-mail: ayis@ayis.org.

Research reported in this publication is part of the Medical Imaging Data Resource Center and was made possible by the National Institute of Biomedical Imaging and Bioengineering of the National Institutes of Health under contracts 75N92020C00008 and 75N92020C00021. In addition, this research has been funded by the University of Illinois at Chicago Center for Clinical and Translational Science (CCTS) award ULTR002003. Drs Pyrros and Siddiqui were funded by MIDRC; and Drs Rodríguez-Fernández and Galanter were funded by UIC. The authors state that they have no conflict of interest related to the material discussed in this article. Drs Pyrros and Siddiqui are partners and all other authors are employees.

Copyright © 2021 American College of Radiology

1546-1440/21/$36.00 ■ https://doi.org/10.1016/j.jacr.2021.09.010

Conclusions: A CNN model was predictive of aortic atherosclerosis in two cohorts (one ambulatory and one hospitalized) with COVID-19. The discrepancy between the CNN model and the administrative code, Δ_{vasc}, was associated with language preference in the ambulatory cohort; in the hospitalized cohort, this discrepancy was associated with social deprivation index. The absence of administrative code(s) was associated with Δ_{vasc} in the combined cohorts, suggesting that Δ_{vasc} is an independent predictor of health disparities. This may suggest that biomarkers extracted from routine imaging studies and compared with electronic health record data could play a role in enhancing value-based health care for traditionally underserved or disadvantaged patients for whom barriers to care exist.
Key Words: Deep learning, vascular disease, value-based health care, health disparities, hierarchical condition categories

J Am Coll Radiol 2022;19:184-191. Copyright © 2021 American College of Radiology

INTRODUCTION

Comorbidities are strongly associated with increased severity of coronavirus disease 2019 (COVID-19), disproportionately affecting racial and ethnic minorities [1]. Many factors contribute to these disparities, including socioeconomics, geography, environment, and health care accessibility, all potentially resulting in underdiagnosis and undertreatment of disease [2]. Racial/ethnic minority populations tend to have a greater degree of underlying comorbidities, which can increase the risk for mortality from COVID-19 [2-4]. Non-White patients hospitalized with COVID-19 were more likely to have a higher severity of infection on admission chest radiographs (CXRs), associated with limited English proficiency [5]. Detecting and measuring health disparities remains difficult and complex [6] and requires new technologies and techniques in a multidisciplinary approach.

In tandem with an increasing recognition of structural health disparities within the US health care delivery system, transformative progress toward value-based health care (VBH) is occurring. VBH is a data-dependent operational construct that emphasizes improved outcomes and decreased costs by managing chronic comorbidities, with reimbursements proportional to disease burden [7]. CMS maintains specific ICD10 codes, hierarchical condition categories (HCCs), that have predictive power for future hospital costs [8]. HCCs contain groupings of multiple ICD10 codes, generated through encounters with health care providers and recorded in administrative data. These data elements are often more reproducible and amenable to analysis than manual review of electronic health records (EHRs). These administrative data also predict mortality in patients with COVID-19 [9]. Using a convolutional neural network (CNN) to connect HCCs to CXRs can convert the images into useful biomarkers of patients' chronic disease burden [10].

Deep learning (DL) has been extensively documented to propagate health care disparities and biases, mostly through the use of biased training data, limiting its generalizability [11]. Conversely, it is possible to use DL algorithms to detect such disparities. We chose thoracic atherosclerosis, most commonly identified by calcification of the aortic knob, as our primary feature. Although the visual detection of thoracic atherosclerosis is trivial, the systemic nature of atherosclerosis and its association with multiple disease processes (cardiac, renal, peripheral arterial, cerebrovascular disease, and diabetes) make it relevant as a potential biomarker. Using a CNN-based DL classifier [10] on CXRs of patients with COVID-19, we can predict the presence of vascular disease. This prediction can then be compared with administrative data to determine the discrepancy between the classifier's prediction and the presence or absence of the administrative code for vascular disease.

We hypothesize that this discrepancy is associated with factors that change the interaction of patients with the health care system, which may alter administrative practices and ultimately coding. Regression can be used to find any association of demographic, racial, and socioeconomic factors with the discrepancy between the prediction and administrative data.

METHODS

Study Population

This retrospective study was approved by the institutional review board and was granted waivers of the requirement to obtain informed consent at the institutions at which the two cohorts were based.

There were two cohorts in this study. The first validation cohort (internal ambulatory COVID-19+, n = 814) was seen between March 14, 2020, and October 24, 2020, and had positive real-time reverse transcription polymerase chain reaction COVID-19 test results in the ambulatory or immediate care setting at Duly Health and Care, a large multispecialty group in the suburbs of Chicago. To evaluate nonacute findings related to chronic medical conditions, the search for frontal CXRs was expanded from April 26, 2018, to October 23, 2020, in this ambulatory COVID-19+ cohort, because the development of thoracic atherosclerotic vascular disease is a lengthy process.

The second cohort (external hospitalized COVID-19+, n = 485) was seen at a large urban tertiary academic hospital in Chicago, the University of Illinois Hospital, between March 14, 2020, and August 12, 2020, and underwent

frontal chest radiography in the emergency department and had positive real-time reverse transcription polymerase chain reaction COVID-19 test results.

Image Acquisition and Analysis

CXRs for the ambulatory COVID-19+ cohort were obtained conventionally using digital posteroanterior radiography (no portable radiographs). CXRs for the hospitalized COVID-19+ cohort were all portable. All CXRs were extracted from a PACS using a scripted method (SikuliX version 2.0.2) and saved as deidentified 8-bit grayscale portable network graphics files (ambulatory cohort) or 24-bit Joint Photographic Experts Group files (hospitalized cohort).

DL CXR Classifier

A CNN-based DL classifier was used to produce an estimate of the likelihood of vascular disease (administrative code HCC-108). This tool has been described previously [10] and was developed on patients such as those in the ambulatory cohort, over a retrospective period of 10 years. All of the CXRs were analyzed by this tool using a high-resolution portable network graphics or Joint Photographic Experts Group file. The result is an estimate of the likelihood of the presence of a code in HCC-108 category (atherosclerotic vascular disease) ranging from 0 to 1. Occlusion-based attribution maps, in which areas of the image are occluded to quantify how the model's prediction changes for the class [12], were generated as a sanity check (Captum version 0.3.1).

Clinical Data

Clinical variables included sex, age, self-reported race/ethnicity, language preference, body mass index, and history of vascular disease as determined by HCC codes from the EHR and administrative data. For patients who did not self-report, race/ethnicity data were imputed using geolocation and surname from US census data [13]. Self-reported races were categorized as Black or Hispanic and all others for the purposes of modeling.

Social Deprivation Index

To control for geographic health inequities, we imputed the publicly available social deprivation index (SDI) by referencing the associated ZIP code tabulation areas [14]. The SDI is based on the American Community Survey and is used "to quantify levels of disadvantage across small areas, evaluate their associations with health outcomes, and address health inequities" [15]. The SDI is a metric that combines demographic data of poverty, high school dropouts, renting, overcrowding, lack of car ownership, and unemployment into a granular ZIP code–level ranking. The SDI, together with other measures, can be used to identify areas that may need additional health care resources.

Consensus Interpretation

Expert interpretations of CXRs were provided by two board-certified radiologists (A.P. and N.S.) with 11 and 10 years of posttraining experience, respectively, for the presence or absence of thoracic aortic atherosclerosis in the ambulatory cohort. Both radiologists were blinded to the results of the DL classifier or any clinical characteristics. Cohen's κ coefficient was calculated to measure interrater reliability of the two radiologists, and cases of disagreement were reconciled by consensus. The CXRs from the hospitalized cohort were not interpreted by the radiologists because of HIPAA limitations.

Statistical Analysis

Demographic characteristics, clinical findings, and CXR DL outcomes were compared between the internal ambulatory and external hospitalized cohorts using two-sided χ^2 tests and t tests. Models for each cohort were generated to evaluate the classifier's predictions of vascular disease against the ground truth (administrative data), using a receiver operating characteristic curve in which the area under the curve was calculated. The classifier's predictions of vascular disease for the ambulatory cohort were further evaluated against the radiologists' reading. Confidence intervals (CIs) and comparison of receiver operating characteristic curves were produced using the method of DeLong et al [16].

The numeric difference between the presence or absence of the administrative code for vascular disease (HCC-108) and the classifier's predictions of vascular disease was defined as Δ_{vasc} and ranged between -1 and $+1$. Multivariate linear regression was performed to examine the association of age, sex, race/ethnicity, language preference, and SDI. Linear model β coefficients, R^2 values, and P values were generated. We further evaluated the associations of Δ_{vasc}, age, sex, race/ethnicity, language preference, and SDI with the likelihood of having none versus one or more HCC codes using logistic regression and generated odds ratios (ORs) and CIs for these associations. $P < .05$ were considered to indicate statistical significance, and analysis was conducted in R version 4.0.2 (R Foundation for Statistical Computing, Vienna, Austria).

RESULTS

Patient Characteristics

A total of 1,299 patients were included in this study, 814 from the internal ambulatory COVID-19+ cohort and 485 from the external hospitalized COVID-19+ cohort (Fig. 1, Table 1). Participants in the hospitalized cohort compared with the ambulatory cohort were more likely to be older (51 versus 63 years of age, $P < .001$), Black (8% versus 48%, $P < .001$), or Hispanic (8% versus 48%, $P < .001$) and to have a non-English-language preference (8% versus 25%, $P < .001$) and a higher mean SDI (31 versus 88, $P < .001$) (Fig. 2). Race was imputed for 42 patients (5%) in the

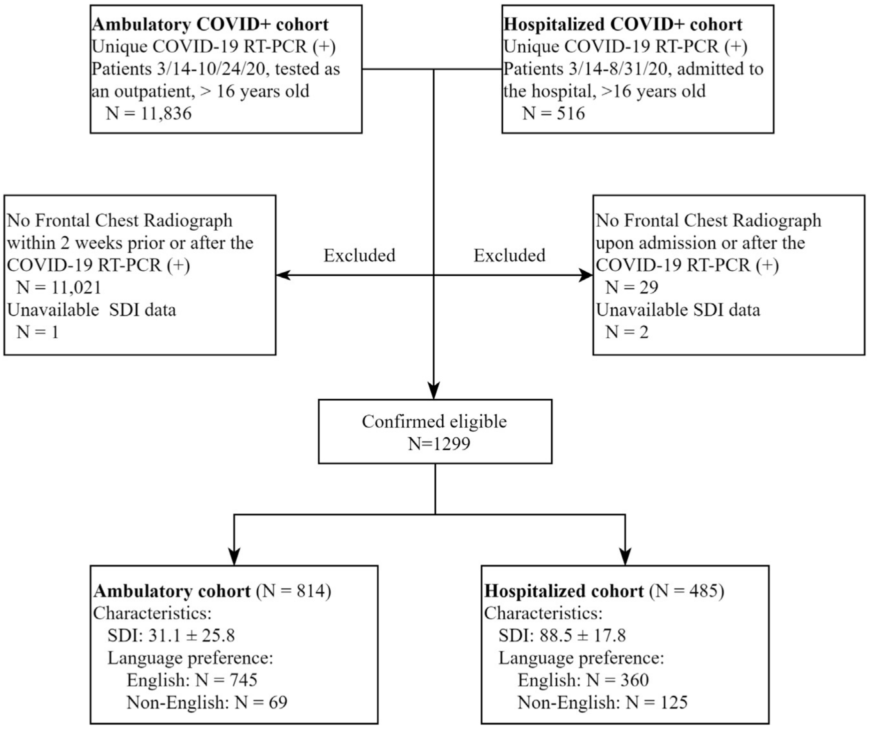

Fig. 1. Flowchart of patient inclusion per cohort. Patients with no or negative real-time reverse transcription polymerase chain reaction (RT-PCR) test results, patients who did not undergo chest radiography, and patients with no social deprivation index (SDI) information were excluded. A total of 1,299 patients were eligible for this study. COVID-19 = coronavirus disease 2019.

ambulatory cohort and 6 patients (1.2%) in the hospitalized cohort. The hospitalized cohort had a higher prevalence of vascular disease compared with the ambulatory cohort (21% versus 8%). The DL classifier predicted both older age and a higher index of vascular disease in the hospitalized cohort compared with the ambulatory cohort, findings that were both consistent with the EHR data. A model evaluating the classifier's predictions for vascular disease compared with the EHR-based administrative data (HCC-108) showed areas under the curve of 0.851 (95% CI, 0.816-0.887) in the ambulatory cohort and 0.694 (95% CI, 0.641-0.748) in the hospitalized cohort, with $P < .001$, using DeLong's method [16]. The relationship between the classifier's predictions for vascular disease and the radiologists' consensus interpretation had an area under the receiver operating characteristic curve of 0.89 (95% CI, 0.86-0.92), with the radiologists having a Cohen's κ of 0.92, demonstrating strong agreement.

Figure 3 shows representative frontal CXRs from the ambulatory (Fig. 3A) and hospitalized (Fig. 3B) cohorts, demonstrating how the DL model analyzed the radiographs and generated the likelihoods of vascular disease.

Modeling of Δ_{vasc}

As shown in Table 2, linear regression modeling of Δ_{vasc} in the ambulatory cohort demonstrated significant associations with age, sex, Black or Hispanic race/ethnicity, and non-English-language preference but no significant association with SDI. In the hospitalized cohort, the significant associations were with SDI and age.

The likelihood of having none versus one or more HCC codes was associated with Δ_{vasc} (OR, 0.336; 95% CI, 0.209–0.538; $P < .001$), age (OR, 1.059; 95% CI, 1.049-1.069; $P < .001$), Black or Hispanic race/ethnicity (OR, 1.576; 95% CI, 1.124-2.210; $P < .01$), and non-English-language preference (OR, 1.738; 95% CI, 1.170-2.583; $P < .01$) (Table 3).

DISCUSSION

In this study we adapted a previously published CNN DL model to identify the presence of thoracic atherosclerotic disease from frontal CXRs and then combined these results with EHR administrative data from two cohorts with linear models. We found the CNN DL classifier to be predictive of vascular disease, validated in two disparate COVID-19 cohorts. The prediction of vascular disease was associated with multiple demographic findings of age, sex, self-reported race/ethnicity, language preference, and ZIP code–based SDI, which is a proxy for poverty and social disparities strongly associated with reduced health and health care access. It is often difficult to understand exactly what an image-based CNN is using to make a prediction. Occlusion mapping can be used to visualize the portion of the image

Table 1. Demographics, clinical findings, and convolutional neural network CXR characteristics per cohort

Characteristic	Ambulatory (n = 814)	Hospitalized (n = 485)	*P*
Age (y)	50.8 ± 16.2	56.3 ± 16.4	<.001
Sex			.554
Male	389 (47.8)	240 (49.5)	
Female	425 (52.2)	245 (50.5)	
Race/ethnicity			<.001
White	562 (69)	27 (5.6)	
Black	65 (8)	234 (48.2)	
Hispanic	124 (15.2)	220 (45.4)	
Asian	63 (7.7)	4 (0.8)	
Language preference			<.001
English	745 (91.5)	360 (74.2)	
Non-English	69 (8.5)	125 (25.8)	
SDI	31.1 ± 25.7	88.5 ± 17.8	<.001
BMI (kg/m^2)	30.8 ± 7.08*	32.2 ± 10.1	.089
Vascular disease diagnosis (EHR HCC-108)	72 (8.8)	105 (21.6)	<.001
Patients without any HCC codes	455 (56)	88 (18)	
DL model predictions using frontal CXR			
Predicted age (y)	54.2 ± 13.6	60.7 ± 10.4	<.001
Vascular disease (HCC-108) probability output[†]	0.254 ± 0.231	0.413 ± 0.212	<.001

Note: Data are expressed as mean ± SD or as number (percentage). BMI = body mass index; CXR = chest radiograph; DL = deep learning; EHR = electronic health record; HCC = hierarchical condition category; SDI = social deprivation index.
*Fourteen participants did not have recorded BMI in the ambulatory cohort.
[†]Normalized probability from 0 to 1 of vascular disease output by the DL classifier.

that is most important for the likelihood of the diagnosis. The occlusion mapping in our cohort demonstrates positive attribution to the cardiovascular structures for vascular disease (Fig. 3).

Our data demonstrated a discrepancy between the CXR classifier and the EHR-based administrative code for vascular disease. There were significant associations with race/ethnicity, SDI, and language preference, which varied in our two socioeconomically and ethnically diverse cohorts. This discrepancy was associated with higher SDI values in a cohort with a higher mean SDI but not in a more affluent, lower risk cohort with a much lower mean SDI. This may

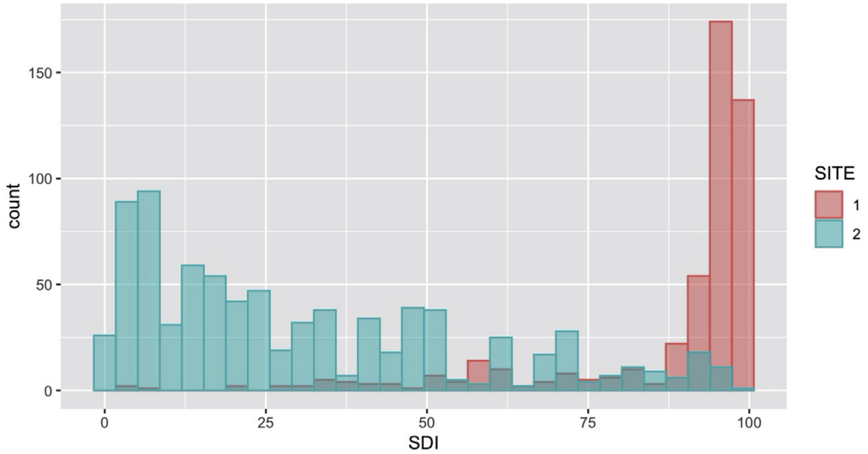

Fig. 2. Histogram distribution of the social deprivation index (SDI) across two sites (site 1, hospitalized cohort; site 2, ambulatory cohort) showing a bimodal distribution and wide separation of the two cohorts.

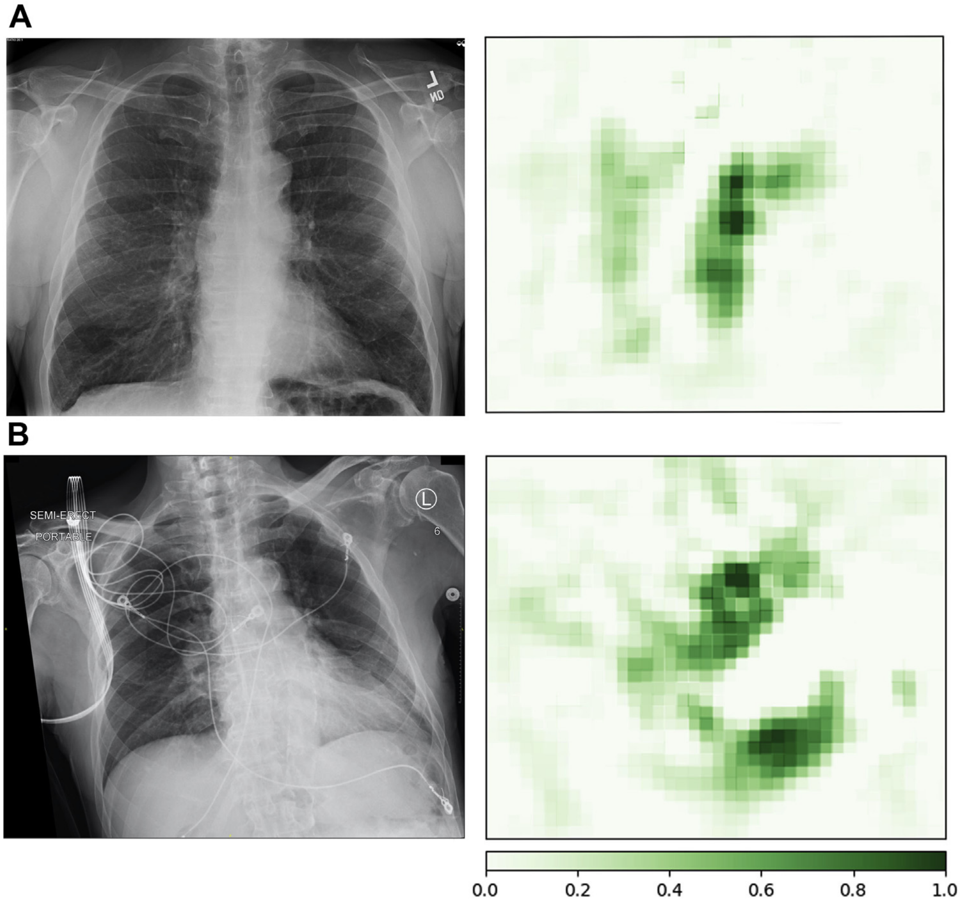

Fig. 3. Occlusion maps with the input chest radiograph on the left and output positive attribution map on the right. The darker green areas, when occluded from the image, positively affect the model's prediction more significantly, representing a larger number on the scale. Occlusion maps for the prediction of vascular disease in a 63-year-old White male patient (A) without an associated electronic health record diagnosis code from the ambulatory cohort and an 86-year-old Black male patient (B) also without a diagnosis of vascular disease from the hospitalized cohort. Positive attributions primarily relate to the aorta, with calcified atherosclerotic visible at the aortic knob.

mean that social deprivation must reach a certain level threshold before it affects coding discrepancy.

We found that the likelihood of having any administrative (HCC) code was associated with vascular diagnosis discrepancy Δ_{vasc}, age, Black or Hispanic race/ethnicity, and non-English-language preference (Table 3). Although there are many unmeasured variables, this suggests that it is possible that the discrepancies in administrative codes are associated with socioeconomic and ethnic factors.

As we transition to a more value-based model for health care delivery and reimbursement, it will be increasingly important to extract the maximum possible administrative codes from available data, because cost-effective care and institutional profitability are tightly linked in VBH [17]. Extracting data from a radiograph and feeding through a trained model to identify "at risk" patients who might benefit from extra clinical, ancillary, and administrative attention may help meet VBH management metrics and improve overall patient care and institutional reimbursement. We offer this work as a proof of concept, with specific model improvements left as future work for interested investigators.

Traditionally, comorbidities have been obtained through patient history and medical records, but there are known racial disparities in provider-patient informing of incidental medical findings [14]. In addition, there are known linguistic barriers, such as speaking only a non-English language, that have been magnified during the COVID-19 pandemic [18]. Although aortic atherosclerosis is exceedingly common with advanced age [19], the discrepancy in its reporting may indicate more important health disparities. DL algorithms are deterministic, meaning that they will produce the same result for the same image, whereas radiologists have stochastic elements in their results, which is why we used Cohen's κ to evaluate interobserver variability in the radiologists' interpretations. Cohen's κ between the two radiologists was very strong at 0.92 but was not perfect.

Table 2. Modeling the difference between the convolutional neural network–predicted and administrative prevalence of vascular disease (Δ_{vasc})

Characteristic	Ambulatory		Hospitalized	
	Coefficient	*P*	Coefficient	*P*
Age	0.005	<.001	0.003	<.01
Sex	0.044	<.05	NA	NS
Black or Hispanic race/ethnicity	−0.048	<.05	NA	NS
Non-English language preference	−0.083	<.05	NA	NS
SDI	NA	NS	0.002	<.05
Adjusted R^2	.11		.022	
P	<.001		0.0201	

Note: NA= not applicable; SDI = social deprivation index.

If underserved patients most at risk for poor health outcomes are similarly most at risk for failure in provider-patient informing and missing information because of language limitations, can a radiologic study fill the gap? We believe so. Automated notification of treating physicians by EHR, text message, or email could be implemented to alert them that a patient, on the basis of the imaging findings, might have undocumented pathologies that warrant further investigation.

It is interesting that the larger the discrepancy factor Δ_{vasc}, the more likely there were no codes present (Table 3). This might be completely normal and expected for young adults in their 20s, but our cohorts had mean ages in the 50s. We live in a global, hypermobile world where people of many different ethnicities, national origins, and economic means may present for care. For example, a 55-year-old non-English-speaking refugee might present de novo for the first time in a US medical care setting with numerous barriers in access to care. Δ_{vasc}, and its association with a lack of HCC coding documentation, may indicate a potential “tip of the iceberg” situation, with more extensive undocumented and likely undertreated pathology lurking under an initial presentation, such as COVID-19 in this setting.

This is relevant not only for the rare John Doe patient but also in cases of demographic information missing from the chart by error, patient inability to provide a history, or inability to obtain data from prior institutions. Imaging biomarkers extracted from CXRs offer unique opportunities to identify undocumented, underdiagnosed, or undiagnosed illnesses in high-risk patients because they are relatively common and inexpensive tests, frequently performed either around or at admission [20,21]. Moreover, imaging biomarkers can alert the medical staff of underlying medical conditions not previously diagnosed, which helps clinicians take a comprehensive approach to conditions not previously known by regular methods (chart review, history taking, etc). CXRs are almost always obtained on presentation consistent with COVID-19 in the emergency department or immediate care clinics. In addition, such imaging biomarkers may unmask health disparities not readily apparent from other data sources. Our DL model allowed us to make a prediction regarding the probability of thoracic vascular disease as a comorbidity and was correlated with administrative EHR diagnoses. HCC codes are also predictive of repeat admission [22].

Table 3. Associations between none and one or more hierarchical condition category codes for the combined cohort of ambulatory and hospitalized patients, using binomial logistic regression

Characteristic	Odds Ratio	95% Confidence Interval	P
Age	1.059	1.049-1.069	<.001
Ambulatory site (reference hospitalized site)	0.215	0.149-0.312	<.001
Black or Hispanic race/ethnicity (reference white or Asian)	1.576	1.124-2.210	.008
Non-English language preference (reference English language)	1.738	1.169-2.584	.006
Δ_{vasc}	0.336	0.209-0.538	<.001

Our study was limited by several factors. First, we did not perform a manual chart review on our cohorts to assess for additional clinical documentation of associated comorbidities, such as vascular disease. Absent or missing diagnoses may have been lost upon transfer from one health system to another, omitted by provider error, or contained within unstructured EHR data, like clinical notes. Although a limitation, this is one of the factors we reviewed by looking at the difference between DL-estimated disease and administrative data. Last, the implementation of DL models remains a technical challenge for many institutions and practices, with relatively few data collection standards or standards of algorithmic development and a lack of widespread adoption. Although we showed the predictive power of the DL classifier when using the hospitalized portable CXRs, the CNN was not trained on portable films.

In conclusion, DL techniques have a well-deserved reputation for propagating biases in medicine. Still, here we show how they can help mitigate these biases, in particular detecting thoracic vascular disease that may be a biomarker for at-risk patients with poor SDI scores, of non-White ethnicity, or with a non-English language preference. Clearly, a multitude of barriers can exist. Critically, this approach performs independently of any additional clinical data, permitting use when patient history and examination information are unknown or difficult to obtain.

TAKE-HOME POINTS

- A DL CXR classifier was predictive of thoracic atherosclerotic vascular disease in patients with COVID-19 compared with the presence of the administrative code for vascular disease in EHRs.
- The discrepancy between the classifier predictions and coded vascular disease demonstrated significant associations with race/ethnicity, SDI, and language preference. These discrepancies were associated with an at-risk cohort with higher mean SDI but not a more affluent cohort with lower mean SDI. This may mean that social deprivation must surpass a threshold before it has an impact. Furthermore, absence of coded vascular disease, even when controlling for age, sex, site, race/ethnicity, and language, was itself suggested as a potential marker for underdiagnosis or underdocumentation.

ACKNOWLEDGMENTS

The authors thank Melinda Willis and Monica Harrington for editing and reviewing this manuscript. The authors also thank Patrick Cole and Andrew Chen for their DL expertise.

REFERENCES

1. Richardson S, Hirsch JS, Narasimhan M, et al. Presenting characteristics, comorbidities, and outcomes among 5700 patients hospitalized with COVID-19 in the New York City area. JAMA 2020;323:2052-9.
2. Webb Hooper M, Nápoles AM, Pérez-Stable EJ. COVID-19 and racial/ethnic disparities. JAMA 2020;323:2466-7.
3. Williamson EJ, Walker AJ, Bhaskaran K, et al. Factors associated with COVID-19-related death using OpenSAFELY. Nature 2020;584:430-6.
4. Yang J, Zheng Y, Gou X, et al. Prevalence of comorbidities and its effects in patients infected with SARS-Cov-2: a systematic review and meta-analysis. Int J Infect Dis 2020;94:91-5.
5. Joseph NP, Reid NJ, Som A, et al. Racial and ethnic disparities in disease severity on admission chest radiographs among patients admitted with confirmed coronavirus disease 2019: a retrospective cohort study. Radiology 2020;297:E303-12.
6. Ward JB, Gartner DR, Keyes KM, Fliss MD, McClure ES, Robinson WR. How do we assess a racial disparity in health? Distribution, interaction, and interpretation in epidemiological studies. Ann Epidemiol 2019;29:1-7.
7. Brady AP, Bello JA, Derchi LE, et al. Radiology in the era of value-based healthcare: a multi-society expert statement from the ACR, CAR, ESR, IS3R, RANZCR, and RSNA. Radiology 2021;298:486-91.
8. Juhnke C, Bethge S, Mühlbacher AC. A review on methods of risk adjustment and their use in integrated healthcare systems. Int J Integr Care 2016;16:4.
9. King JT Jr, Yoon JS, Rentsch CT, et al. Development and validation of a 30-day mortality index based on pre-existing medical administrative data from 13,323 COVID-19 patients: the Veterans Health Administration COVID-19 (Vaco) index. PLoS ONE 2020;15:e0241825.
10. Pyrros A, Flanders AE, Rodríguez-Fernández JM, et al. Predicting prolonged hospitalization and supplemental oxygenation in patients with COVID-19 infection from ambulatory chest radiographs using deep learning. Acad Radiol 2021;28(8):1151-8.
11. Straw I. The automation of bias in medical artificial intelligence (AI): decoding the past to create a better future. Artif Intell Med 2020;110:101965.
12. Captum: model interpretability for PyTorch. Available at: https://captum.ai/. Accessed June 30, 2021.
13. Who are you? Bayesian prediction of racial category using surname and geolocation website Updated May 17, 2021. Available at: https://github.com/kosukeimai/wru. Accessed June 30, 2021.
14. Schut RA. Racial disparities in provider-patient communication of incidental medical findings. Soc Sci Med 2021;277:113901.
15. Robert Graham Center. Social deprivation index (SDI) website. Available at: https://www.graham-center.org/rgc/maps-data-tools/sdi/social-deprivation-index.html. Accessed June 30, 2021.
16. DeLong ER, DeLong DM, Clarke-Pearson DL. Comparing the areas under two or more correlated receiver operating characteristic curves: a nonparametric approach. Biometrics 1988;44:837-45.
17. Henkel RJ, Maryland PA. The risks and rewards of value-based reimbursement. Front Health Serv Manage 2015;32:3-16.
18. Ortega P, Martínez G, Diamond L. Language and health equity during COVID-19: lessons and opportunities. J Health Care Poor Underserved 2020;31:1530-5.
19. Gallino A, Aboyans V, Diehm C, et al. Non-coronary atherosclerosis. Eur Heart J 2014;35:1112-9.
20. Kerr IH. The preoperative chest X-ray. Br J Anaesth 1974;46:558-63.
21. American College of Radiology. ACR Appropriateness Criteria: routine chest radiography. Available at: https://acsearch.acr.org/docs/69451/Narrative/. Accessed September 26, 2021.
22. Mosley DG, Peterson E, Martin DC. Do hierarchical condition category model scores predict hospitalization risk in newly enrolled Medicare advantage participants as well as probability of repeated admission scores? J Am Geriatr Soc 2009;57:2306-10.
