# Supplementary material for: Validation of a deep learning, value-based care model to predict mortality and comorbidities from chest radiographs in COVID-19
Source: PLOS Digit Health. 2022 Aug 1;1(8):e0000057. doi: 10.1371/journal.pdig.0000057 (PMC9931278; doi:10.1371/journal.pdig.0000057)
Supplement: S2 File — Pyrros A, Flanders AE, Rodríguez-Fernández JM, Chen A, Cole P, Wenzke D, Hart E, Harford S, Horowitz J, Nikolaidis P, Muzaffar N, Boddipalli V, Nebhrajani J, Siddiqui N, Willis M, Darabi H, Koyejo O, Galanter W. Predicting Prolonged Hospitalization and Supplemental Oxygenation in Patients with COVID-19 Infection from Ambulatory Chest Radiographs using Deep Learning. Acad Radiol. 2021 Aug;28(8):1151–1158. doi: 10.1016/j.acra.2021.05.002. Epub 2021 May 21. PMID: 34134940; PMCID: PMC8139280. (PDF) [file pdig.0000057.s002.pdf]

Preliminary Investigation

Predicting Prolonged Hospitalization and Supplemental Oxygenation in Patients with COVID-19 Infection from Ambulatory Chest Radiographs using Deep Learning

Ayis Pyrros, MD, Adam Eugene Flanders, MD, Jorge Mario Rodríguez-Fernández, MD, Andrew Chen, MS, Patrick Cole, MS, Daniel Wenzke, MD, Eric Hart, MD, Samuel Harford, MS, Jeanne Horowitz, MD, Paul Nikolaidis, MD, Nadir Muzaffar, Viveka Boddipalli, MD, Jai Nebhrajani, MD, Nasir Siddiqui, MD, Melinda Willis, MPH, Houshang Darabi, PhD, Oluwasanmi Koyejo, PhD, William Galanter, MD, PhD

Rationale and Objectives: The clinical prognosis of outpatients with coronavirus disease 2019 (COVID-19) remains difficult to predict, with outcomes including asymptomatic, hospitalization, intubation, and death. Here we determined the prognostic value of an outpatient chest radiograph, together with an ensemble of deep learning algorithms predicting comorbidities and airspace disease to identify patients at a higher risk of hospitalization from COVID-19 infection.

Materials and Methods: This retrospective study included outpatients with COVID-19 confirmed by reverse transcription-polymerase chain reaction testing who received an ambulatory chest radiography between March 17, 2020 and October 24, 2020. In this study, full admission was defined as hospitalization within 14 days of the COVID-19 test for $>$ 2 days with supplemental oxygen. Univariate analysis and machine learning algorithms were used to evaluate the relationship between the deep learning model predictions and hospitalization for $>$ 2 days.

Results: The study included 413 patients, 222 men (54%), with a median age of 51 years (interquartile range, 39−62 years). Fifty-one patients (12.3%) required full admission. A boosted decision tree model produced the best prediction. Variables included patient age, frontal chest radiograph predictions of morbid obesity, congestive heart failure and cardiac arrhythmias, and radiographic opacity, with an internally validated area under the curve (AUC) of 0.837 (95% CI: 0.791−0.883) on a test cohort.

Conclusion: Deep learning analysis of single frontal chest radiographs was used to generate combined comorbidity and pneumonia scores that predict the need for supplemental oxygen and hospitalization for $>$ 2 days in patients with COVID-19 infection with an AUC of 0.837 (95% confidence interval: 0.791−0.883). Comorbidity scoring may prove useful in other clinical scenarios.

Keywords: COVID-19; deep learning; multi-task learning; convolutional neural networks; chest radiography.

© 2021 The Association of University Radiologists. Published by Elsevier Inc. All rights reserved.

Abbreviations: AUC area under curve, **BMI** body mass index, **CHF** congestive heart failure, **CI** confidence interval, **CNN** convolutional neural network, **COPD** chronic obstructive pulmonary disease, **COVID-19** coronavirus disease 2019, **CXR** chest radiograph, **EHR** electronic health record, **HCC** hierarchical condition category, **ICD10** International Classification of Diseases, Tenth Revision, **MTL** multi-task learning, **ROC** receiver operating characteristic, **RT-PCR** reverse transcription-polymerase chain reaction

Acad Radiol 2021; ■:1–8

From the DuPage Medical Group, Radiology (A.P., N.M., V.B., N.S., M.W.); Thomas Jefferson University Hospital, Radiology (A.E.F.); University of Illinois at Chicago, Department of Neurology (J.M.R.-F.); University of Illinois at Urbana-Champaign, Department of Computer Science (A.C., P.C., O.K.); NorthShore University HealthSystem Research Institute, Department of Radiology (D.W.); University of Illinois at Chicago, Mechanical and Industrial Engineering (S.H., H.D.); Northwestern Memorial Hospital, Northwestern University, Radiology (E.H., J.H., P.N.); University of Illinois at Chicago, Department of Medicine (J.N., W.G.). Received March 30, 2021; revised April 28, 2021; accepted May 2, 2021. Address correspondence to , 40 S Clay St, Hinsdale, IL 60521. e-mail: ayis@ayis.org

© 2021 The Association of University Radiologists. Published by Elsevier Inc. All rights reserved.
https://doi.org/10.1016/j.acra.2021.05.002

INTRODUCTION

The coronavirus disease 2019 (COVID-19) pandemic placed unprecedented demand on healthcare systems. Although many infected individuals have mild or no symptoms, some become very ill and may be hospitalized for long durations (1). Comorbid conditions like diabetes and cardiovascular disease are associated with more severe cases of COVID-19 (2). Unfortunately, relevant comorbidities are sometimes unknown or unrecognized by the medical provider and patient, limiting the provider's ability to perform a proper risk assessment (3). Currently, the extraction of comorbidity data is based on contemporaneously provided patient history, manual record review, and/or electronic health record (EHR) queries (4), and the results are imperfect and often incomplete. The purpose of this study was to develop a deep learning algorithm that could predict the likely presence of relevant comorbidities, in combination with an algorithm to quantify opacity, from frontal chest radiographs (CXRs), and thereby enable providers to more effectively risk-stratify patients presenting with COVID-19 infection.

COVID-19 infection is diagnosed with reverse transcription-polymerase chain reaction (RT-PCR) or antigen tests. In patients with limited symptoms, additional testing is often unnecessary. In patients with higher risk for severe disease or complications, however, including those presenting with more severe symptoms, chest radiography is widely used for evaluation (5).

The Centers for Medicare and Medicaid Services uses a specific subset of hierarchical condition category (HCC) codes from the International Classification of Diseases, Tenth Revision (ICD10) to model chronic disease comorbidities and their associated costs of care for value-based payment models (6). The codes are generated through encounters with healthcare providers and recorded in administrative (billing) data. These data elements are often more reproducible and more amenable to query as compared to broader searching of EHR systems. Using a convolutional neural network (CNN) to link these categorical codes to a CXR can convert the images into useful biomarker proxies for a patient's chronic disease burden. For instance, a high categorical prediction for HCC18 would indicate that a CXR strongly suggests diabetes with chronic complications.

Multiple predictive clinical models of the course of COVID-19 infection use demographic information, clinically obtained comorbidity data, laboratory markers, and radiography (7,8). Radiography is used as a proxy for infection severity by quantifying the geographic extent and degree of lung opacity (7,8). However, we are not aware of previous models using radiographs to directly predict or quantify comorbidities that contribute to patient outcomes. We hypothesize that an ensemble CNN model derived from frontal CXRs, composed of comorbidities and geographic—opacity scores, can predict prolonged hospitalization and supplemental oxygenation of ambulatory COVID-19 patients.

METHODS

This study was approved by the institutional review board and was granted waivers of Health Insurance Portability and Accountability Act authorization and written informed consent.

Patient Cohort and Clinical Setting

The two cohorts in this study comprise patients receiving an outpatient frontal CXR at DuPage Medical Group, a large multi-specialty group in the suburbs of Chicago, IL. The first cohort consists of 14,121 CXRs of patients from 2010 to 2019, who were enrolled in the Medicare Advantage program. These patients had CXRs for typical clinical indications, like pneumonia, chest pain, and cough, and none of these patients had COVID-19 infection. This cohort was used to develop and validate a multi-task CNN to predict HCC-based comorbidities.

The second cohort was seen between March 17, 2020 and October 24, 2020 and received both a CXR and a positive RT-PCR COVID-19 test in the ambulatory or immediate care setting. Some of the patients went to the emergency department after the positive RT-PCR test and some were hospitalized. The EHR clinical notes were reviewed for information regarding the reason, date of admission, treatments, and length of hospitalization in days. This cohort is called COVID +. We define "full admission" as hospitalization > 2 days with supplemental oxygen.

In cases of multiple positive COVID-19 RT-PCR tests, or negative and then positive tests, the first positive test was used as the reference date. Likewise, in patients with multiple outpatient CXRs, the radiograph closest to the initial positive RT-PCR was used, with only one radiograph used per subject. Patients without locally available or recent CXRs, radiographs obtained > 14 days after positive RT-PCR testing, and subjects < 16 years old at the time of radiography were excluded. Patients admitted for > 2 days within 14 days of the RT-PCR test and 7days of chest radiography were defined as full admissions (Fig 1).

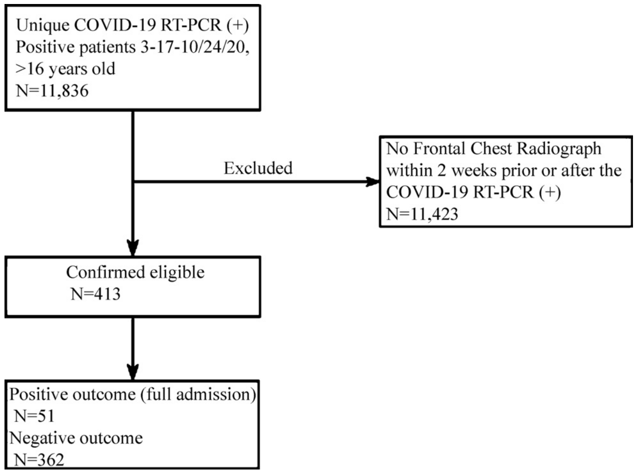

Figure 1. Flow diagram of retrospective cohort study. COVID-19 = coronavirus disease 2019, RT-PCR = reverse transcription-polymerase chain reaction.

Image Acquisition and Analysis

All radiographs were obtained conventionally with digital posteroanterior radiography; no portable radiographs were included. All CXRs were extracted from the PACS system utilizing a scripted method (SikuliX, 2.0.2) and saved as de-identified 8-bit grayscale portable network graphics (PNG) files.

Deep Learning

A multi-task CNN was trained on anonymized outpatient frontal CXRs from 2010 to 2019 randomly split into 80% training and 20% test data sets. The gold standard was the EHR ICD10 codes. The 80% set was trained on sex, age, and six common ICD10 HCC codes (model v23, used by the Centers for Medicare and Medicaid Services). ICD10 codes were obtained via queries of the EHR (Epic) from the transactions table. The following HCC categories were used: diabetes with chronic complications (HCC18), morbid obesity (HCC22), congestive heart failure (CHF; HCC85), specified heart arrhythmias (HCC96), vascular disease (HCC108), and chronic obstructive pulmonary disease (COPD; HCC111). In the training set, each radiographic file was a separate row, with the absence of any associated ICD10 HCC codes labeled as 0, and the presence of one or more codes labeled as 1.

Binary cross-entropy was used as the objective function in PyTorch (version 1.01; pytorch.org), and the Adam optimizer (9) with a learning rate of 0.0005 to train the neural network. The learning rate was decreased by a factor of 10 when the loss ceased to decrease for 10 iterations. Random horizontal flips (20%), random affine (rotate image by max of 10 degrees), random resized crop scale (range 1.0, 1.1), ratio (range 0.75, 1.33), and random perspective (distortion scale of 0.2, with a probability 0.75) were applied for data augmentation. Image normalization was performed by using the standard PyTorch function, with the mean and standard deviation of the pixel values computed over the training set. For image resizing, we used the PIL library to downscale to 256 × 256 with the Lanczos filter. A customized CoordConv (10) ResNet34 model was pretrained on the CheXpert dataset (11); CoordConv allows the convolution layer access to its own input coordinates, using an extra coordinate channel. The training was performed on a Linux (Ubuntu 18.04; Canonical, London, England) with two Nvidia TITAN GPUs (Nvidia Corporation, Santa Clara, Calif), with CUDA 11.0 (Nvidia) for 50 epochs over 10.38 hours. Training used image and batch sizes of 256 × 256 and 64, respectively. All programs were run in Python (Python 3.6; Python Software Foundation, Wilmington, Del).

Multi-Task Learning (MTL)

MTL is a general framework for learning several tasks simultaneously using their shared structure (12). In contrast to standard (single-task) learning where each task is learned independently, MTL exploits inter-task relationships to improve the representation and prediction quality. MTL can be implemented using various approaches, including explicit parameter sharing and implicit parameter sharing (e.g., using nuclear norm regularization) (13). When individual task performance improves, this is known as “positive transfer” and indicates that joint learning is superior to separate learning. In contrast, though less common, individual task performance can degrade with MTL, a “negative transfer” phenomenon (14). MTL is a well-established approach to machine learning that has been applied in computer vision, natural language processing, and medical applications (14), among others (15).

To quantify the geographic extent and severity of opacity of infection, we used the open-source program COVID-Net (16), which produces two scores: one for the geographic extent and one for the severity of opacity. Both scores were normalized from 0 to 1.

Clinical Data

Clinical variables evaluated included patient age, sex, and length of stay. History of COPD, diabetes, morbid obesity (body mass index [BMI] > 40), CHF, cardiac arrhythmias, or vascular disease was determined by ICD10 ambulatory billing codes. Other than the COVID-19 RT-PCR test, outpatient lab results were not used, as most were unavailable within 24 hours of the COVID-19 RT-PCR positive test.

Statistical Analysis

One-to-one comparison of categorical and continuous variables was done with logistic regression. The t-test was not used as many of the variables were nonparametric. The predictions for the six HCCs were compared to the COVID + cohort billing claims to test that this model, derived from a large cohort of patients prior to the COVID-19 pandemic, had predictive power with the COVID + cohort. The analysis used the area under the curve (AUC) of receiver operating characteristic (ROC) curves. No hypothesis testing was done. Logistic regression produced odds ratios with 95% confidence intervals (CIs). All tests were two-sided, a p value < 0.05 was deemed statistically significant, and analysis was conducted in R version 4 (R Foundation for Statistical Computing, Vienna, Austria).

Fit of Radiographic Features to Outcome Data

The COVID+ cohort had 11 input features and 1 outcome: whether or not a patient had a full admission (> 2 days) within 7 days of the CXR. To assess the contributions and performance of the six-variable HCC CNN model and the two-variable geographic extent and opacity severity model, separate logistic regressions and AUC curves were generated and compared.. Then the data were split into a training/validation set (70%) and a testing set (30%). Several machine learning models, including logistic regression, decision trees, random forest, XGBoost, LightGBM, and CatBoost, were developed and optimized in Python (Python 3.6) using the

TABLE 1. Demographics and Findings of 413 Outpatients.

Characteristics	Full Admission (*n* = 51)		No Full Admission (*n* = 362)		Odds Ratio (CI)	*p* value
Age, Mean (SD)	60.5	(12.9)	48.4	(16.0)		<0.0001
Sex						0.86
Female	23	(45.1)	168	(46.4)		
Male	28	(54.9)	194	(53.6)		
Race						0.47
White	22	(43.1)	200	(55.2)		
Hispanic	12	(23.5)	81	(22.4)		
Other, Non-Hispanic	8	(15.7)	38	(10.5)		
African American	5	(9.8)	22	(6.1)		
Clinical and Radiological Features						
BMI, Mean (SD)	32.4	(6.7)	30.5	(7.1)		0.07
A1C, Mean (SD)	6.4	(1.3)	6.0	(1.1)		0.13
Chest X-ray Opacity, Mean (SD)	0.412	(0.12)	0.337	(0.1)	920 (63–13,600)	<0.0001
Chest X-ray Geo Score, Mean (SD)	0.255	(0.1)	0.21	(0.051)	12,000 (240–60,000)	<0.0001
Variables Predicted by the Multi-Task CNN Model						
Diabetes with Complications HCC18, mean (SD)	0.332	(0.2)	0.161	(0.2)	36.2 (9.7–136)	<0.0001
Morbid obesity HCC22, Mean (SD)	0.226	(0.3)	0.156	(0.2)	2.63 (0.94–7.3)	0.065
CHF HCC85, Mean (SD)	0.222	(0.2)	0.092	(0.1)	33.2 (7.9–140)	<0.0001
Cardiac Arrhythmias HCC96, Mean (SD)	0.174	(0.2)	0.067	(0.1)	48.9 (9.0–265)	<0.0001
Vascular Disease HCC108, Mean (SD)	0.372	(0.2)	0.218	(0.2)	14.1 (4.2–47.8)	<0.0001
COPD HCC111, mean (SD)	0.143	(0.2)	0.075	(0.1)	10.2 (2.2–47.8)	0.0032
Age, Mean (SD)	62.0	(10.1)	51.9	(13.4)	1.05 (1.03–1.08)	<0.0001

A1C, glycated haemoglobin; BMI, body mass index; CHF, congestive heart failure; CI, confidence interval; COPD, chronic obstructive pulmonary disease; COVID-19, coronavirus disease 2019; HCC, hierarchical condition category.

training/validation set (17–22). The model development process uses recursive feature elimination to find the optimal feature set for each model. In this approach, a single feature is removed at each step and the model is evaluated on the validation set. The quality of the fits to the data were measured using the ROC AUC. The best model was tested against the testing set for a final measure of prediction.

RESULTS

Patient Characteristics

In total, 413 patients were included in the COVID+ cohort (Fig 1 and Table 1); 221 were White [53%], 31 were Asian [7%], 96 were Hispanic [23%], 27 were Black [6.5%], and 38 were other or unknown [10%]. Fifty-one (12%) of the patients had a full admission, with all requiring supplemental oxygen, and four died. No deaths were recorded within 48 hours of admission. Their mean age was 60 years (±13, median = 58, range = 39–97), 54% were male, and they had a mean length of stay of 10 days (±9, median = 7, range = 3–45). Patients without a full admission had a mean age of 48 years (±15, median = 49, range = 16–89) and 53% were male.

CNN Analysis

A set of 14,121 anonymized unique frontal CXRs (compliant with the Health Insurance Portability and Accountability Act) was used to train a CNN to predict six HCCs using ambulatory billing data. The CNN was also trained to predict the age of the patient. The mean age of the patients, at the time of the radiograph, was 66 ± 13 years, and 57% of the patients were women. First, a training set of 11,257 (80%) radiographs was used to develop the CNN, which was then tested against a randomly selected set of 2,864 (20%) radiographs.

The CNN produces a probability for each predicted variable including age, diabetes with chronic complications (HCC18), morbid obesity (HCC22), CHF (HCC85), vascular disease (HCC108), and COPD (HCC111). This could be compared to the HCC data for the test cohort. For each variable, the relationship is summarized by a ROC, and results are shown in Table 2.

Because the CNN was trained on a cohort selected from all ambulatory frontal CXRs prior to 2020, we compared the HCC predictions on the COVID+ cohort to determine whether the CNN was predictive in this clinical setting (Table 2). A representative frontal CXR from a COVID-19 patient is shown in Figure 2, which demonstrates how the CNN analyzes the radiographs and generates the likelihoods of comorbidities. All saliency maps were generated in Python, with the integrated gradients attribution algorithm, which computes the gradient integrals of the output prediction for the class index, with respect to the input image pixels (23). Importantly, this technique does not modify the CNN model.

TABLE 2. Multi-task CNN HCC-Based Comorbidity Predictions from a Randomized Test Cohort (n = 2,864) of Frontal Chest Radiographs, Compared to a Cohort of COVID-19 Patients (n = 413), based on EHR data.

Variable	Disease Description	CNN Test Cohort		COVID+ Cohort		
		AUC	95% CI	AUC_{COVID}	95% CI	EHR HCCCount*
HCC18	Diabetes with Chronic Complications	0.798	0.780−0.816	0.765	0.694−0.836	44 (10.7%)
HCC22	Morbid Obesity	0.927	0.912−0.942	0.910	0.878−0.948	39 (9.4%)
HCC85	Congestive Heart Failure	0.850	0.834−0.867	0.836	0.744−0.929	11 (2.7%)
HCC96	Specified Heart Arrhythmias	0.837	0.816−0.857	0.750	0.657−0.862	19 (4.6%)
HCC108	Vascular Disease	0.729	0.711−0.747	0.868	0.823−0.912	30 (7.3%)
HCC111	Chronic Obstructive Pulmonary Disease	0.836	0.818−0.854	0.845	0.709−0.981	7 (1.7%)
Total AUC (All Codes)		0.856	0.850−0.862	0.850	0.820−0.879	150 (6%)

AUC, area under the curve; CNN, convolutional neural network; COVID-19, coronavirus disease 2019; HER, electronic health record; HCC, hierarchical condition category.

* The EHR HCC count represents the number of unique patients with the HCC code in the COVID+ cohort.

Imaging Characteristics of the COVID+ Cohort

We used the COVID-Net deep learning model (16) to quantify the extent and degree of opacity, which generates geographic and opacity (geographic−opacity) scores, normalized from 0 to 1. For full admission, the average geographic scores were 0.26 ± 0.01 (median = 0.22), and average opacity scores were 0.41 ± 0.12 (median = 0.39), while for those without full admission, scores were 0.21 ± 0.05 (median = 0.19) and 0.34 ± 0.08 (median = 0.31), respectively, as shown in Table 1.

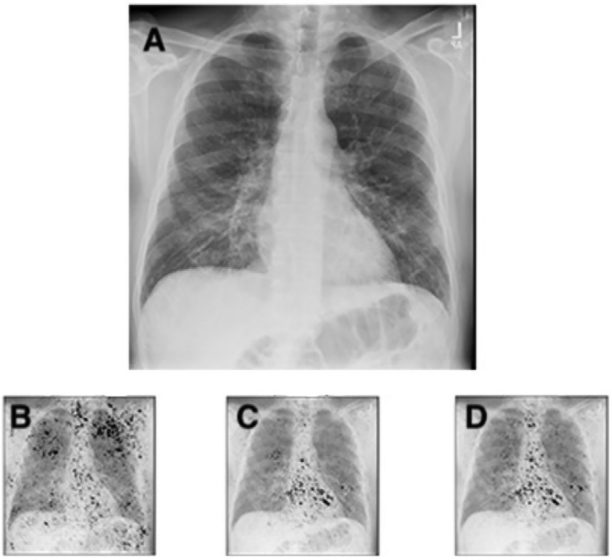

Figure 2. Chest radiograph (a) of a 63-year-old male patient with COVID-19 hospitalized for 7 days, and with a BMI of 26, demonstrating subtle ground glass opacities in a lower lung distribution, with increased geographic (0.34) and opacity scores (0.64). The integrated gradients saliency maps, with darker shades representing higher scores from the multi-task comorbidity HCC model: morbid obesity (HCC22; B), CHF (HCC85; C), cardiac arrhythmias (HCC96; D). Much of the activation seen is outside the lung parenchyma, with notable activation of the axillary soft tissue for obesity (b), and heart for CHF and cardiac arrhythmias (c, d). The activations for CHF and cardiac arrhythmias are very similar, but demonstrate subtle differences, with slightly greater activation at the left atrium and aortic knob (d), suggesting the associations of vascular disease and atrial fibrillation. BMI = body mass index, CHF = congestive heart failure, COVID-19 = coronavirus disease 2019, HCC = hierarchical condition category.

Univariate and Multivariate Analysis

Table 1 shows information on the COVID+ cohort divided by presence or absence of a full admission. Patients with full admissions were significantly older (mean age, 60 years ± 13 vs. 48 years ± 15, $p < 0.0001$). Univariate analysis demonstrated our frontal CXR comorbidity model predictions for diabetes with chronic complications, cardiac arrhythmias, CHF, COPD, predicted age, and geographic extent and severity of opacity were all significant predictors ($p < 0.05$), while morbid obesity was not.

Binary classification logistic regressions of the predicted six-variable CNN HCC model, geographic−opacity model, and combined eight-variable model were plotted as ROC curves (Fig 3), excluding patient age, against the entire COVID+ cohort. The fit for the HCC model had an AUC of 0.768 (95% CI: 0.708−0.829), the geographic−opacity model had an AUC of 0.693 (95% CI: 0.611−0.776), and the combined model had an AUC of 0.796 (95% CI: 0.734−0.859). The AUC for just the binomial EHR-based ICD10 HCC codes was 0.5646 (95% CI: 0.507−0.6221). Utilizing the method of DeLong to compare the AUCs of the combined and geographic−opacity regression models demonstrated a statistically significant difference, with a P value of 0.001.

The patient's age, comorbidities predicted by the CNN, and airspace disease (geographic−opacity scores) measured by the COVID-Net deep learning model (6) were used to model the likelihood of a full admission. A development cohort (n = 216) and validation cohort (n = 73) were first used to produce models using different methods, and these were tested against a 30% test cohort (n = 124) (models outlined in methods). The two best methods for prediction on

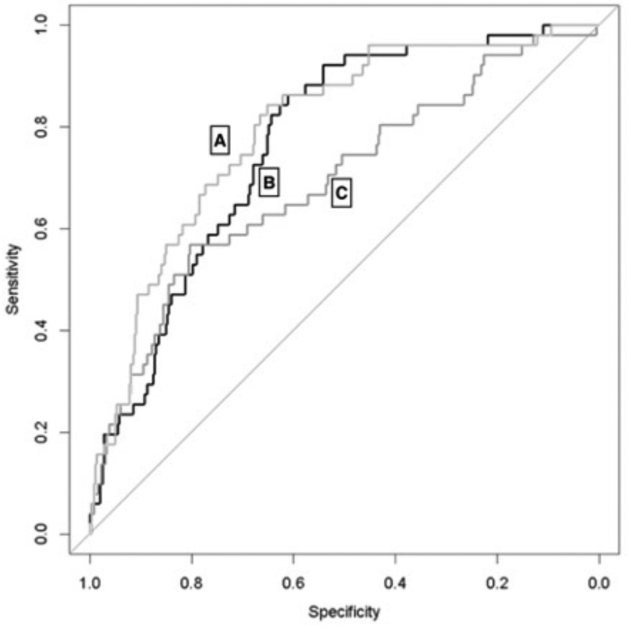

Figure 3. ROC curves from binary classification logistic regressions of the combined HCC and geographic–opacity models (A, light grey, AUC = 0.796 (95% CI: 0.734–0.859), six HCC comorbidities (B, black, AUC = 0.768, 95% CI: 0.708–0.829), and geographic–opacity CNN scores (C, dark grey, AUC = 0.693, 95% CI: 0.611–0.776) for the prediction of prolonged hospitalization with oxygen supplementation. AUC = area under the ROC curve, CNN = convolutional neural network, HCC = hierarchical condition category, ROC = receiver operating characteristic.

the development/validation cohort were logistic regression (17) (AUC 0.81) and XGBoost (20) (AUC 0.94). The XGBoost model was then used to model the remaining independent 30% test cohort. The final fit had an AUC of 0.837 (95% CI: 0.791–0.883). This model required five variables for prediction; the five variables used were age, opacity, and CNN-derived HCCs for morbid obesity, CHF, and specified heart arrhythmias.

DISCUSSION

In this preliminary study we developed an ensemble deep learning model to predict supplemental oxygenation and hospitalization of > 2 days in outpatients testing positive for COVID-19. This model was based only on patient age and a conventional outpatient frontal CXR image obtained before admission in 413 patients and showed an AUC of 0.837 (95% CI: 0.791–0.883), with a boosted random forest method. There is a complementary benefit of the ensemble deep learning models when predicting comorbidities and predicting geographic extent and severity of opacity on CXRs, as demonstrated by comparison of the AUCs.

There are numerous clinical models of outcomes in COVID-19, many focused on admitted and critically ill hospitalized patients (24). Several models have utilized the CXR as a predictor of mortality and morbidity for hospitalized COVID-19 patients, based on the severity, distribution, and extent of lung opacity present (24). ICD10 administrative data have similarly been used to effectively predict mortality in COVID-19 patients (25). To our knowledge, no published studies have utilized features of the CXR other than those related to airspace disease to make a prognostic prediction in COVID-19. This deep learning technique adds value when assessing patients with unknown medical history or awaiting laboratory testing. A significant number of COVID-19 patients demonstrate little to no abnormal lung opacity on initial radiographic imaging (26), and comorbidity scoring is beneficial in these patients when infection might be in the early stages. Additionally, comorbidity scoring could be helpful in identifying patients who could benefit from earlier initiation of treatment such as antibody therapy or close clinical surveillance.

Even before advanced deep learning techniques, CXRs have been shown to correlate with the risk of stroke, vascular resistance, and atherosclerosis through the identification of aortic calcification (27–29). Similar deep learning methods were used on two large sets of frontal CXRs and demonstrated predictive power for mortality (29). In another study (30), deep learning was used on a large public data set to train a model to predict age from a frontal CXR. These studies all suggest that the CXR can serve as a complex biomarker.

Our deep learning techniques allowed us to make predictions regarding the probabilities of comorbidities such as morbid obesity, diabetes, CHF, arrhythmias, vascular disease and COPD. Although these results do not replace traditional diagnostic methods (i.e., glycated hemoglobin, BMI), we did find that they were predictive using the gold standard of ICD10 HCC administrative codes, with all the AUC confidence intervals well above 0.5, demonstrating a predictive value. When tested on an entirely different cohort, the COVID+ patients, the prediction similarly demonstrated AUCs well above 0.5, even in those with low opacity scores. Lastly, when using binary classification logistic regression from the combination of CNN models on the COVID+ cohort, we see a 'lift' of the combined model AUC, with a statistically significant difference ($p = 0.001$).

These HCC predictions were combined with quantitative predictions on lung opacity and patient age, and the resulting model had discriminatory ability in predicting which ambulatory patients would require full admission within 14 days of the positive RT-PCR test. Interestingly, morbid obesity was not significant in the univariate analysis but was significant in the multivariate analysis, suggesting Simpson's paradox, where the correlation is changed when the variables are combined (31). The final model used three of the HCC predictions to help classify patients, and thus, even if the predictions are not entirely mapped to a comorbidity, the resulting measurement strongly correlates and has significance for hospitalization due to COVID-19.

In this study on ambulatory patients, a minority had timely and complete laboratory assessments, unlike hospitalized patients who typically undergo extensive testing at presentation, limiting our ability to use laboratory results. As

Schalekamp et al. described, many of these laboratory markers are not widely available or are expensive (8).

The use of comorbidity indices derived from frontal CXRs has many potential benefits. In many acute clinical settings, comorbidities may be undocumented or unknown at the time of presentation (3). The amount of time needed to take a full history can sometimes be an impediment, especially with high patient volumes seen during the pandemic. Since a CXR is a frequent part of the initial assessment of a COVID-19 patient, the predicted comorbidity scores could be rapidly available for all patients. Additionally, the EHR provides a predominately binomial system of documenting disease (present or absent), but not all patients have the same burden of disease, as in diabetes for instance. It is possible that a model like this one could help distinguish these differences.

Many of the models used for the prognosis of COVID-19-positive patients look at patients already hospitalized and attempt to predict clinical deterioration, intensive care unit admission, death, or some combination. When internally tested on their own data, the AUCs reported in the literature can be over 0.9 (32) but often do not use an independent test set, which increases the risk of overfitting and obtaining an artificially high AUC. Numerous models have AUCs lower than 0.8 (33), with most models ranging between 0.8 and 0.9 (25). These models are often dependent on the EHR data to make their predictions, but missing data from the EHR can adversely model predictions, meaning a model from a single source like CXRs can add significant value (34). Although we cannot directly compare our study to these others because of the differences in patient populations and study design, we believe our predictive power is comparable with those of other prognostic studies, and our model uses only the patient age and the information in the frontal CXR.

Our study was limited to our integrated healthcare system and its geographical distribution and is only internally validated at this time. In many cases, we had limited access to the patient's complete hospitalization records and laboratory assessments, restricting endpoint analysis. In the early parts of the pandemic, many patients underwent computed tomography chest imaging in lieu of CXR, because of the limited availability of RT-PCR testing. Additionally, many patients had imaging at other locations, which were not available in this study. Artificial intelligence models typically demonstrate poorer performance when used in other settings, due to different patient demographics and equipment. No portable radiographs were used in the training or testing of our model, which could limit its use in emergency departments and hospitals. Lastly, implementation of artificial intelligence models remains a technical challenge at most institutions and practices, with relatively few available platforms or widespread adoption.

CONCLUSION

We found that a MTL deep learning model of comorbidities and geographic extent and severity of opacity was predictive of prolonged hospitalization and supplement oxygenation based on a single outpatient frontal CXR. This result suggests that further validation and extension of this particular methodology is warranted.

REFERENCES

1. Huang C, Wang Y, Li X, et al. Clinical features of patients infected with 2019 novel coronavirus in Wuhan, China. The Lancet. 2020;395 (10223):497–506. doi: 10.1016/S0140-6736(20)30183-5
2. Richardson S, Hirsch JS, Narasimhan M, et al. Presenting characteristics, comorbidities, and outcomes among 5700 patients hospitalized with COVID-19 in the New York City area. JAMA 2020; 323(20):2052. doi:10.1001/jama.2020.6775.
3. Yang J, Zheng Y, Gou X, et al. Prevalence of comorbidities and its effects in patients infected with SARS-Cov-2: a systematic review and meta-analysis. Int J Infect Dis 2020; 94:91–95. doi:10.1016/j.ijid.2020.03.017.
4. Sparks R, Salskov AH, Chang AS, et al. Pocket change: a simple educational intervention increases hospitalist documentation of comorbidities and improves hospital quality performance measures. Qual Manag Health Care. 2015; 24(2):74–78.
5. Kim HW, Capaccione KM, Li G, et al. The role of initial chest X-ray in triaging patients with suspected COVID-19 during the pandemic. Emerg Radiol 2020: 1–5. doi:10.1007/s10140-020-01808-y.
6. Juhnke C, Bethge S, Mühlbacher AC. A review on methods of risk adjustment and their use in integrated healthcare systems. Int J Integr Care 2020; 16(4). doi:10.5334/ijic.2500.
7. Toussie D, Voutsinas N, Finkelstein M, et al. Clinical and chest radiography features determine patient outcomes in young and middle-aged adults with COVID-19. Radiology 2020; 297(1):E197–E206. doi:10.1148/radiol.2020201754.
8. Schalekamp S, Huisman M, van Dijk RA, et al. Model-based prediction of critical illness in hospitalized patients with COVID-19. Radiology 2021; 298(1):E46–E54. doi:10.1148/radiol.2020202723.
9. Kingma DP, Ba J. Adam: a method for stochastic optimization. arXiv:14126980 [cs] [Internet]. 2017 [cited 2020 Nov 17]; Available at: http://arxiv.org/abs/1412.6980
10. Liu R, Lehman J, Molino P, et al. An intriguing failing of convolutional neural networks and the CoordConv solution. arXiv:180703247 [cs, stat] [Internet]. 2018 [cited 2020 Nov 17]; Available at: http://arxiv.org/abs/1807.03247
11. Irvin J, Rajpurkar P, Ko M, et al. CheXpert: a large chest radiograph dataset with uncertainty labels and expert comparison. arXiv:190107031 [cs, eess] [Internet]. 2019 [cited 2020 Nov 17]; Available at: http://arxiv.org/abs/1901.07031
12. Caruana R. Multitask learning: a knowledge-based source of inductive bias. In: In: Proceedings of the Tenth International Conference on International Conference on Machine Learning (ICML'93, Amherst, MA, USA. Morgan Kaufmann Publishers Inc.; 1993:41–48.
13. Argyriou A, Evgeniou T, Pontil M. Convex multi-task feature learning. Mach Learn 2008; 73(3):243–272. doi:10.1007/s10994-007-5040-8.
14. Harutyunyan H, Khachatrian H, Kale DC, Ver Steeg G, Galstyan A. Multitask learning and benchmarking with clinical time series data. Scientific Data 2019; 6(1):96. doi:10.1038/s41597-019-0103-9.
15. Collobert R, Weston J. A unified architecture for natural language processing: deep neural networks with multitask learning. In: In: Proceedings of the 25th International Conference on Machine Learning - ICML '08, Helsinki, Finland. ACM Press; 2008:160–167. doi:10.1145/1390156.1390177.
16. Wang L, Wong A. COVID-Net: a tailored deep convolutional neural network design for detection of COVID-19 cases from chest x-ray images. arXiv:200309871 [cs, eess] [Internet]. 2020 [cited 2020 Nov 17]; Available at: http://arxiv.org/abs/2003.09871
17. Hosmer DW, Lemeshow S, Sturdivant RX. Applied Logistic Regression. 3rd edition Hoboken, New Jersey: Wiley, 2013:528.
18. Safavian SR, Landgrebe D. A survey of decision tree classifier methodology. IEEE Transactions on Systems, Man, and Cybernetics 1991; 21 (3):660–674. doi:10.1109/21.97458.
19. Liaw A, Wiener M. Classification and regression by randomForest. R News 2002; 2(3):18–22.

20. Chen T, Guestrin C. XGBoost: a scalable tree boosting system. In: In: Proceedings of the 22nd ACM SIGKDD International Conference on Knowledge Discovery and Data Mining (KDD ’16), San Francisco, California, USA. Association for Computing Machinery; 2016:785–794. doi:10.1145/2939672.2939785.
21. Ke G, Meng Q, Finley T, et al. LightGBM: a highly efficient gradient boosting decision tree. In: NIPS'17: Proceedings of the 31st International Conference on Neural Information Processing Systems, Red Hook, NY, USA. Curran Associates, Inc.; 2017:3146–3154.
22. Prokhorenkova L, Gusev G, Vorobev A, Dorogush AV, Gulin A. CatBoost: unbiased boosting with categorical features. In: NIPS’18: Proceedings of the 32nd International Conference on Neural Information Processing Systems, Montréal, Canada. Curran Associates, Inc.; 2018:6639–6649.
23. Sundararajan M, Taly, A, Ya Q. Axiomatic attribution for deep networks. arXiv: 1703.01365 [CoRR][Internet]. 2018 [cited 2021 Jan 5]; Available at: http://arxiv.org/abs/1703.01365
24. Wynants L, Calster BV, Collins GS, et al. Prediction models for diagnosis and prognosis of COVID-19: systematic review and critical appraisal. BMJ 2020; 369:m1328. doi:10.1136/bmj.m1328. [Internet].
25. King Jr JT, Yoon JS, Rentsch CT, et al. Development and validation of a 30-day mortality index based on pre-existing medical administrative data from 13,323 COVID-19 patients: the veterans health administration COVID-19 (VACO) index. PLoS One. 2020; 15(11):e0241825doi:10.1371/journal.pone.0241825.
26. Pan F, Ye T, Sun P, et al. Time course of lung changes at chest CT during recovery from coronavirus disease 2019 (COVID-19). Radiology 2020; 295(3):715–721. doi:10.1148/radiol.2020200370.
27. Adar A, Onalan O, Keles H, Cakan F, Kokturk U. Relationship between aortic arch calcification, detected by chest x-ray, and renal resistive index in patients with hypertension. Med Princ Pract 2019; 28(2):133–140. doi:10.1159/000495786.
28. Kim YS, Park HY, Yun K-H, Park H, Cheong JS, Ha YS. Association of aortic knob calcification with intracranial stenosis in ischemic stroke patients. J Stroke 2013; 15(2):122–125. doi:10.5853/jos.2013.15.2.122.
29. Lu MT, Ivanov A, Mayrhofer T, Hosny A, Aerts HJWL, Hoffmann U. Deep learning to assess long-term mortality from chest radiographs. JAMA Netw Open 2019; 2(7):e197416doi:10.1001/jamanetworkopen.2019.7416.
30. Karargyris A, Kashyap S, Wu JT, Sharma A, Moradi M, Syeda-Mahmood T. Age prediction using a large chest x-ray dataset. arXiv:190306542 [cs] [Internet]. 2019 [cited 2020 Nov 17]; Available at: http://arxiv.org/abs/1903.06542
31. Hernán MA, Clayton D, Keiding N. The Simpson’s paradox unraveled. Int J Epidemiol 2011; 40(3):780–785. doi:10.1093/ije/dyr041.
32. Ma X, Ng M, Xu S, et al. Development and validation of prognosis model of mortality risk in patients with COVID-19. Epidemiol Infect 2020; 148: e168. doi:10.1017/S0950268820001727.
33. Yu C, Lei Q, Li W, et al. Clinical characteristics, associated factors, and predicting COVID-19 mortality risk: A retrospective study in Wuhan, China. Am J Prev Med 2020; 59(2):168–175. doi:10.1016/j.amepre.2020.05.002.
34. Agor J, Özaltın OY, Ivy JS, Capan M, Arnold R, Romero S. The value of missing information in severity of illness score development. J Biomed Inform 2019; 97:103255.
