# Supplementary material for: Validation of a deep learning, value-based care model to predict mortality and comorbidities from chest radiographs in COVID-19
Source: PLOS Digit Health. 2022 Aug 1;1(8):e0000057. doi: 10.1371/journal.pdig.0000057 (PMC9931278; doi:10.1371/journal.pdig.0000057)
Supplement: S1 Table — Sample ICD10 hierarchical condition category (HCC) codes map to their respective HCC categories. In this example, the patient has codes for both diabetes with chronic complication and without complications; the hierarchy means HCC18 supersedes HCC19, and the lower coefficient is dropped. In addition, there is a diagnosis for congestive heart failure (CHF), which results in a disease interaction raising the RAF score by an additional coefficient. Lastly, having more than one ICD10 code per category does not alter the coefficients. *In our deep learning model, we excluded the demographic component to separately control for age and sex. (DOCX) [file pdig.0000057.s003.docx]

**S1 Table:** A representative example demonstrating how the risk adjustment factor (RAF) is calculated for a female patient aged 65–69 years. Sample ICD10 hierarchical condition category (HCC) codes map to their respective HCC categories. In this example, the patient has codes for both diabetes with chronic complication and without complications; the hierarchy means HCC18 supersedes HCC19, and the lower coefficient is dropped. In addition, there is a diagnosis for congestive heart failure (CHF), which results in a disease interaction raising the RAF score by an additional coefficient. Lastly, having more than one ICD10 code per category does not alter the coefficients. *In our deep learning model, we excluded the demographic component to separately control for age and sex.

| Sample ICD10 HCC Diagnosis Codes | Variable | Description | Community, NonDual, Age Coefficients |
| --- | --- | --- | --- |
|  | 65–69 female* | Demographics | 0.321 |
| E08.21 | HCC18 | Diabetes with chronic complications | 0.305 |
| E13.9 | HCC19 | Diabetes without complication | ~~0.105~~ |
| Z68.41 | HCC22 | Morbid obesity | 0.244 |
| B33.24 | HCC85 | Congestive heart failure | 0.337 |
|  | Diabetes_CHF | Disease interaction | 0.119 |
|  | Total RAF |  | 1.366 |
