# Supplementary material for: Validation of a deep learning, value-based care model to predict mortality and comorbidities from chest radiographs in COVID-19
Source: PLOS Digit Health. 2022 Aug 1;1(8):e0000057. doi: 10.1371/journal.pdig.0000057 (PMC9931278; doi:10.1371/journal.pdig.0000057)
Supplement: Attachment — Submitted filename: PLOS-Response_to_Reviewer.docx [file pdig.0000057.s005.docx]

PDIG-D-22-00013
Validation of a Deep Learning, Value-Based Healthcare Model to Predict Mortality and Comorbidities from Chest Radiographs in COVID-19
PLOS Digital Health

Dear Editor,

Thank you for giving us the opportunity to submit a revised draft of our manuscript titled “Deep Learning–Based Digitally Reconstructed Tomography of the Chest in the Evaluation of Solitary Pulmonary Nodules: A Feasibility Study” to *PLOS Digital Health.* We appreciate the time and effort that you and the reviewers have dedicated to providing valuable feedback on our manuscript. We have been able to incorporate changes to reflect the suggestions provided by the reviewers. We have highlighted the changes within the manuscript. Here is a point-by-point response to the reviewers’ comments and concerns.

**Response to Reviewers**

**Reviewer #1**

Major:

1. The methods were not detailed enough for others to reproduce their works. I don’t understand the role of feature selection. What’s the task by which the AUC was calculated? I am worried about the data leakage problem occuring in this step. Did they use a standalone data split for feature selection? They also mentioned the use of multivariable logistic regression and backward elimination with fractional polynomial transformation to include the variables from the DL. What is the purpose and process of backward elimination? What are the results if they don’t go through this step?
   a. *Response: Feature selection in the deep learning portion of the model is through MTL and AUC performance. Specifically, for the deep learning model, we started with the 10 most common conditions (ICD-HCC codes) available in the EHR and based on their performance (AUC ROC) selected the best six-predictors. We have attached our source code (python pytorch) in a link above.*
   b. *Feature selection in the LR model occurs separately. Our wording was confusing; we have updated it and thank the reviewer for his attention to detail. We used standard train/test/validation splits segregating our data appropriately to avoid training to the test/data leakage and have included that in our report. The “backward elimination with fractional polynomial transformation” was specifically used to develop logistic regression model from the DL predictors. This is well explained in this article, which we have added as a reference to the paper:*
      i. For example, in logistic regression model, covariates are assumed to be linearly associated with response variable in logit scale. However, it is not always the case and the assumption may be wrong. For example, lactate is associated with mortality outcome, but the relationship is not linear ([1](https://www.ncbi.nlm.nih.gov/pmc/articles/PMC4876277/#r1)). Quadratic or cubic terms can be added to an explanatory variable to account for the non-linearity relationship. However, this requires subject-matter knowledge to determine the form of a variable. In exploratory study, such knowledge is always lacking and investigators have to rely on data to determine the functional form. Multivariable fractional polynomial (MFP) method is such a method that it allows software to determine whether an explanatory variable is important for the model, and its functional form ([2](https://www.ncbi.nlm.nih.gov/pmc/articles/PMC4876277/#r2),[3](https://www.ncbi.nlm.nih.gov/pmc/articles/PMC4876277/#r3)) [<https://www.ncbi.nlm.nih.gov/pmc/articles/PMC4876277/>*].*
   c. *The elimination of such a step could affect LR model performance, decreasing the AUC of LR model from the DL predictors, but also would mean a more ad hoc and less systematic approach in choosing the best predictors.*
2. What is the task when pretraining the CXR model on CheXpert?
   a. *Response: Thank you for pointing this out. As novel architectures do not have applicable weights for transfer learning, and there is some disagreement among practitioners whether using pretrained weights from imagenet which has RGB images is appropriate for grayscale radiology images, training from scratch on a large dataset of somewhat related images is a good way to get a well-trained network that can then be fine-tuned on a smaller more specific local dataset which might not be able to fully train the deep network on its own. This is somewhat established in the field of radiology AI so we do not belabor the point further.*
3. Did authors split the dataset into training, validation and testing and use a validation set for hyperparameters selection (or feature selection)?
   a. *Response: We have included this schema which was of course used in the training. We did not use the validation set for hyperparameter selection or feature selection. Hyperparameter selection was done by using the Adam optimizer during training/testing, which is routine in DL computer vision papers. The validation set was the 900 patients from the two cohorts.*
4. The authors mentioned that the MTL can exploit the inter-task relationships but I didn’t see such relationships in their results. I am wondering whether there is an improvement from separate learning? I think they need some implementation details but not only general introduction in Method section and some comparison results in Results section to prove that the MTL is helpful.
   a. *Response: Thank you for pointing this out. We concede that most of this information regarding MTL is well established and known to the body of research, implicit in the use of a multi-class classifier. Therefore, this is extraneous verbiage, our point in this paper isn’t to prove that a MTL is better than single-task learners, which is established in other papers, but that by using a multi-task learner we can produce several imaging biomarkers simultaneously, which can be applied in various scenarios, such as COVID-19 mortality. There are numerous papers and courses which describe MTL and meta learners, for example, https://cs330.stanford.edu, which is why we believe it is beyond the scope of this paper.*
5. In addition to AUC, the authors should report the results with the other evaluation metrics (e.g., **recall and precision**) since the patient numbers of each medical condition and mortality are very small.
   a. *Response: Thank you, we agree and have added this to the manuscript as a separate table, Table 3.*
6. I would suggest that the authors give more **explanation on the visualization maps** to see if the model is looking at the meaningful locations.
   a. *Response: Occlusion mapping demonstrates what areas of the image are important to the DL model’s prediction. The map is produced by blocking or occluding segments of the image and seeing how the model’s numeric prediction is changed in a stepwise manner. The resulting maps demonstrate that the DL model finds areas that a radiologist would find important, such as the heart in CHF, lungs in COPD, and the aorta in vascular disease, etc. We have added more details to the discussion.*
7. Similarly, the authors could give more explanation on **Figure 3 [- means Figure 4]**. There are some other high correlation values besides those five variables.
   a. *Clinically, chronic medical conditions tend to co-exist, as seen in the Figure 4 correlation matrix, we have expanded this on the results section for Figure 4. For example, patients who have COPD often have heart disease.*

Minor:

1. In the first part of the Results section. Why was the mean age described here? “The mean age at the time of the radiograph was 66 ± 13.263 years”. I think it should belong to the data description section.
   a. *Response: Thank you, yes we agree. We have created a new section of patient characteristics and consolidated all data accordingly.*
2. Do horizontal flips of the CXR images make sense? Especially when the model is trying to predict congestive heart failure.
   a. *Response: Current thinking on data augmentation for radiology images is that the submission of altered images functions as a regularizer (https://pubmed.ncbi.nlm.nih.gov/31777848/) rather than presenting different cases to increase the number of images presented to the algorithm. While data augmentation remains an art, improvement in the algorithm as a result of augmentation is more tied to the use of augmentation than the specific augments, as again, it functions more as a regularizer.*
3. Add information in Figure 4 (or its caption) to directly let the reader know it is for mortality prediction.
   a. *We are not sure if the reviewer was referring to a different figure, however Figure 4 is a correlation matrix, showing the degree of agreement between the DL model’s predictions and the EHR diagnostic codes. It does not refer to mortality, but the correlation between the DL predictions in the internal and external cohorts.*

**Reviewer #2**

1. In the Introduction section the authors make a very logical case for using a DL model to predict comorbidities (and some sociodemographics) from chest X-rays. However, the reasoning to convert this into a mortality predictor is somewhat assumed as a natural consequence of the comorbidity prediction. Consequently, in the rest of the manuscript the authors use a logistic regression model for prognostication. The authors claim that this prognostic model is generalizable since it gave an AUC of 0.84 in the combined (internal and external cohort). I have several concerns with regard to this claim:
   a. I am concerned that the value of AUC reported in the combined cohort is higher than the individual components (0.81 in internal and 0.76 in external). Please check the numbers.
   b. *Response: Thank you, we have confirmed the results. The confidence interval is broad in this model, and when we combine the datasets that the AUC is improved. The internal ambulatory dataset had only four deaths, resulting in a large confidence interval, with overall less disease burden, with more negative outcomes. We also would like to point the reviewer to this line in our discussion:*
      i. “This logistic regression model, when applied to both cohorts demonstrated an increased ROC AUC, as the internal cohort had less disease findings, similarly described by Schalekamp [11].
      ii. *Below is the relevant text reference from Schalekamp:*
          1. Our study population, however, was different and consisted exclusively of patients with severe disease who required hospital admission. Our population had a large proportion that developed critical illness (168 of 356, 47%). It is likely that the discriminatory ability of our model would improve when also applied to outpatients, as those patients would show less disease findings than our study population ([26](https://pubs.rsna.org/doi/full/10.1148/radiol.2020202723#r26),[33](https://pubs.rsna.org/doi/full/10.1148/radiol.2020202723#r33)).
2. The model seems to include repeated information (Table 3). For example, comorbidities like COPD, CHF and Diabetes have ORs that indicate a protective role against mortality. I think this is happening because the RAF score already includes information on comorbidities and thus including each comorbidity again in the full model isn't the way to approach this.
   a. *Response: Thank you, we agree that the RAF is a combination of the other comorbidities, including the six included in the DL model. However, the calculated RAF score we used includes all 83 HCCs codes, which are derived from 9,500 ICD10 codes. As we stated, we excluded gender and age from the score since we separately controlled for this in the DL model’s predictions. We have clarified this in our manuscript.*
   b. *Second, we used adjusted ORs, which would be interpreted as the percent change in the odds corresponding to a 1-unit increase in X while holding all other predictors fixed. However, if the logarithm of the odds against the predictor X has a U shape (both low and high values have large odds of the outcome relative to the intermediate values) and the model assumes a linear (straight line) pattern, which could generate the results observed. [https://www.ahajournals.org/doi/10.1161/circulationaha.106.682658]*
3. I think that the prognostic model needs to be re-specified. Why not use all elements of the RAF score and the predicted sociodemographics to run a machine learning model (RF, SVM or XGBoost to predict mortality? Also do this separately for the internal and external cohorts (please don't combine them).
   a. *Response: Thank you for this comment. It is unclear to us if the reviewer is specifically referring to using the EHR data as the predictors or just the DL CXR model predictions. Undoubtedly, there are numerous ways to model the DL predictions. Unfortunately, in the internal ambulatory cohort there were too few cases of mortality (n=4) to produce a reasonable model, with the resulting confidence intervals being very large.*
   b. *An alternative methodology would be to combine the cohorts and split them into training and test data sets, 80/20, with 10-fold cross validation. We have done that below using regularized discriminant analysis, gradient boosting, and support vector machines in R using the caret library.*
   c.

   d. *The above lattice plot shows the performance of these various models on the resampled data set. In this scenario, all three techniques result in similar performance to the logistic regression model we present in the manuscript, with the RDA demonstrating an ROC AUC of 0.83, GBM ROC AUC of 0.81, and SVM ROC AUC of 0.79.*
   e. *Regardless, we believe that the important point here is to demonstrate the usefulness of imaging biomarkers. At this point in the COVID pandemic our specific LR model would not be very helpful, because of intervening vaccines and COVID variants. Instead, we are trying to demonstrate that using ICD10 data in combination with chest radiographs can create imaging biomarkers which can be rapidly applied to various scenarios. We concede if our intent was to find the best model possible to help care for COVID patients presently, applying these additional techniques may be beneficial, but we would need an entirely new dataset.*
4. The authors have modified the ResNet34 (CoordConv) model to improve the classification performance for prediction of the comorbidities. However, in the entire manuscript there is no direct comparison of the performance of the standard ResNet34 with the improved model that the authors used. I suggest that the model accuracy of ResNet34 be compared against that of this improved model.
5. Extending this line of thinking, the authors also need to compare the performance of other CNN architectures – deeper ResNets (101, 152), DenseNet (at least 121), EfficientNets (at least B1), Inception (v3) and Xception. It is possible that the performance of some of these candidate alternatives may be better than the architecture used by the authors. If not, then all the reason to use the authors’ model!
   a. *Response: Thank you for your valuable feedback. Respectfully, we intend this paper to explain the use of imaging biomarkers adapted to predict specific outcomes in COVID. The architecture choice was influenced by compute resource availability, i.e. GPU memory. We concede, that while it is reasonably likely that larger architectures could alter or improve model performance, as could hyperparameter selection, this would change the focus of our paper; from clinical application of value-based imaging biomarkers, and towards model and hyperparameter selection for the purposes of algorithmic comparisons. We feel this is a separate publication best left for future work.*
6. Numbers reported in Table 2 indicate that there was substantial overfitting for some conditions (CHF, COPD, Vascular disease and somewhat for diabetes). The authors need to describe whether this potential overfitting was despite measures like regularization and dropouts? If not, the authors need to add these (and other) measures to reduce overfitting and thus improve the classification performance in the external dataset.
   a. *Response: Thank you, we believe there may be some confusion regarding table 2. In Table 2, the headers reflected an earlier draft which had confusing language, interchangeably using validation and cohort. As this was confusing, and incorrectly implied we were using the validation set in a standard train/validation/test split, we corrected this heading. We appreciate your bringing this confusing terminology to our attention.*
7. The manuscript needs to be copyedited for typos.
   a. *Response: Thank you for this feedback, we have proofread the manuscript again. Please let us know if you see any additional errors.*
